# Evaluation of stress markers in horses during hippotherapy sessions in comparison to being ridden by beginners

**DOI:** 10.1017/awf.2023.6

**Published:** 2023-01-30

**Authors:** Julie FN Potier, Vanessa Louzier

**Affiliations:** 1 The Liphook Equine Hospital, Hampshire, UK; 2 APCSe Agressions Pulmonaires et Circulatoires dans le Sepsis, Université de Lyon, VetAgro Sup – Campus Vétérinaire de Lyon, 1 Avenue Bourgelat, 69280, Marcy-l’Étoile, France

**Keywords:** adrenocorticotropic hormone, animal welfare, cortisol, hippotherapy, horse, stress

## Abstract

Hippotherapy has been used for decades and its benefits to human patients have largely been proven, whether being applied to those with physical or mental disabilities. There have been a plethora of animal welfare studies recently, pertaining especially to ridden horses. This study aimed to investigate stress markers in horses during hippotherapy sessions to address the ethical considerations raised by using horses for therapy. A ridden stress ethogram was established and validated specifically for this study via subjective observation and video recording of a ridden session involving intermediate-level riders. The experiment entailed eight healthy horses undergoing two ridden sessions on separate days, one with disabled riders and one with beginners. Several parameters associated with physiological responses to stress were evaluated at rest, such as heart rate, plasma adrenocorticotropic hormone [ACTH], serum and salivary cortisol. These parameters as well as the behavioural stress score from the ethogram scale were measured during both sessions. No significant differences were found between heart rate, plasma ACTH, and stress scores. Serum and salivary cortisol were significantly lower during the hippotherapy session than during the session with beginners. The current study found no evidence of compromised welfare when horses were used as a therapeutic aid during hippotherapy sessions compared to their usual ridden activity. Although these results indicate that hippotherapy may be ethically justified as it benefits humans without causing harm to the horses, the present study was small, and the results should be interpreted with caution.

## Introduction

Animal-assisted therapies have a variety of applications in human medicine (Grandgeorge & Hausberger 2011) and been used and described for over two decades (Stanley-Hermanns & Miller 2002). Equine-related treatments, in particular, have been investigated and although original reviews considered it controversial for use in psychotherapy due to most studies being methodologically flawed especially in their lack of randomised controlled trials between patients (Anestis *et al.* 2014), hippotherapy has proven beneficial in the clinical progress of disabled patients (Koca & Ataseven 2015; Wood & Fields 2021). The improvements have been observed after horseback riding sessions on patients affected with various types of physical and/or mental disabilities. Recent, well-designed, controlled studies and meta-analyses demonstrating benefits include, amongst others, patients affected with: multiple sclerosis (Bronson *et al.* 2010; Vermöhlen *et al.* 2018), cerebral palsy (Zadnikar & Kastrin 2011; Park *et al.* 2014; Martín-Valero *et al.* 2018; Matusiak-Wieczorek *et al.* 2020), Down syndrome (Champagne & Dugas 2010; Portaro *et al.* 2020), postural instabilities (Silkwood-Sherer *et al.* 2012), acute brain injury (Marquez *et al.* 2020), children with mental impairments and developmental delays (Kraft *et al.* 2019), attention deficit and hyperactivity disorder (Oh *et al.* 2018), autism spectrum disorders (Ajzenman *et al.* 2013), post-traumatic stress disorders (Johnson *et al.* 2018; Shelef *et al.* 2019) and schizophrenia (Jormfeldt & Carlsson 2018). Ethical concerns have been voiced in recent years with the need for assessment and perhaps the use of animals for human therapies undergoing regulation (Loeb 2019; Fine & Andersen 2021). With increasing knowledge about animal welfare, behaviour and stress-related pathologies, multiple studies in horses have investigated the effects of working on stress parameters. These parameters include heart rate, and heart-rate variability, cortisol and behaviour (Munsters *et al.* 2012, Kang & Yun 2016; Uldahl *et al.* 2021). Behavioural ethograms have been established to help assess signs of discomfort during ridden sessions (Henry *et al.* 2017; Dyson & Pollard 2020; Haddy *et al.* 2021). Welfare investigations during, in particular, therapeutic riding have also been attempted and subsequent behavioural analyses have not been suggestive of horses experiencing significant levels of stress (Kaiser *et al.* 2006; Mendonça *et al.* 2019; Arrazola & Merkies 2020; Watson *et al.* 2020), nevertheless better understanding of how this horse-human interaction works, substantiated with a variety of behavioural and physiological parameters is required (Fine & Andersen 2021; Hovey *et al.* 2021) and many studies lack a sound research design, including small sample sizes and control groups. Due to the bias in assessing behaviour as a single stress indicator, the need for other potential biological markers of stress in horses has been investigated, e.g. cardiovascular effects of catecholamine release (sympathetic-adrenal-medullary axis), especially heart rate and heart-rate variability, as well as the hypothalamo-pituitary-adrenal axis endocrine stress response (von Lewinski *et al.* 2013; Ferlazzo *et al.* 2020). Good correlation between circulating markers supports analysis of multiple parameters to increase accuracy (Ferlazzo *et al.* 2018a,b,c). Previous findings have tended to suggest the interactions between horses and riders during therapeutic riding not to be deleterious for the horses when mentally able and mentally impaired riders are compared via behavioural analysis and heart parameters (Cravana *et al.* 2021) and other biological parameters over time especially the hypothalamo-pituitary-adrenal axis (Fazio 2013; Ferlazzo *et al.* 2018a,b,c). In order to increase knowledge on horses’ stress status and focus on whether their welfare suffered in regard to use in therapeutic activities, our study aimed to investigate different behavioural and physiological stress indicators during hippotherapy sessions. The goal being to understand whether hippotherapy was indeed more stressful than a beginners’ riding session which functioned as a control group since horses’ physical activity was typically comparable in both sessions.

## Materials and methods

### Ethical statement

This experiment protocol was subjected to examination from the Ethics Committee for animals’ protection of VetAgro Sup - Campus Vétérinaire de Lyon, France. All procedures and protocol design were authorised under agreement number 1512-2 (meeting 18, 2015) and Directive 2010/63/EU of the European Parliament and of the Council of 22 September 2010 on the protection of animals used for scientific purposes (and feed legislation, if appropriate) were adhered to.

### Pre-clinical study: Development of the specific ridden ethogram scale

A personalised ethogram with weighted scores, based on those seen in other studies into the behaviour of ridden horses was devised and is presented in Table 1. Briefly, parameters included each horse’s general attitude (Sénèque *et al.* 2019), the position of the neck, tail movements, position of the ears and mouth (Dyson & Pollard 2021a,b; Torcivia & McDonnell 2021). The grid and weighted scores were established specifically for the experiment according to ridden ethogram scores (Dyson *et al.* 2017) and taking into account the opinions of a physiology expert (PhD); to differentiate horses displaying stress behaviour from those more relaxed. Video analysis of a ridden session took place to enable validation of the ethogram by the physiology expert, ensuring that different individuals obtained scores that were diverse enough to warrant interpretation. Session scores ranged from 0 (no evidence of stress) to 20 (severely distressed) and took place in the equestrian centre; inside an indoor arena familiar to all participants (riders and horses). Riders had attained an intermediate level of experience, thus were able to contest low-level showjumping competitions (Strunk *et al.* 2018). The scores that ended up being associated with each parameter are shown in Table 1, one weighted mark is used per parameter observed.

**Table 1.** Study ethogram showing associated weightings for each parameter

Parameter	Description	Associated score	Horse
Head carriage	Higher than horizontal, constantly shaking	3	
	Higher than horizontal, occasional head flicks (up to 5)	2	
	Horizontal	1	
	Lower than horizontal	0	
Tail position	Strongly maintained against perineal region	3	
	Squishing (more than 5 times)	2	
	Occasional movement (less than 5 times)	1	
	Relaxed	0	
Ears orientation	Facing backwards	3	
	Moving back and forth constantly	2	
	Stiffly forward	1	
	Relaxed on the sides	0	
Mouth	Constantly opened	3	
	Stereotypy (tongue out, teeth grinding or chattering)	2	
	Shut	1	
	Relaxed with occasional chewing	0	
Direction and pace	Speeds up and changes direction	3	
	Tries to come back to midline	2	
	Slows down pace	1	
	Steady pace and direction	0	
Defence	Bucks/kicks overt discomfort	3	
Mounting behaviour	Walks away	2	
	Stands still	0	
Total calculated horse score			

### Experiment

Eight healthy horses comprising six geldings and two mares were utilised, with age ranging from 12 to 21 years. None of the horses were stressed or in pain. Pre-experiment clinical exams were normal with no horses displaying any discernable signs of lameness. Horses were housed together in their usual environment of an open barn (25 × 10 m; length × width) bedded with straw, with constant access to pasture (700 × 400 m). They were fed *ad libitum* dry hay and provided with permanent access to fresh water via a water trough. Horses had previously been ridden weekly by beginners and disabled people for a minimum of six months prior to the start of the experiment. This took place over a 15-day period in early spring, with mild and cloudy weather and an average temperature of 17°C.

Measurements of basal biological markers and clinical examinations were carried out at rest prior to the onset of the study to rule out any abnormalities, especially any hypothalamo-pituitary axis dysfunction or resting cardiac dysrhythmia. The impaired riders (IR) consisted of four women and four men, aged between 19 and 47 years and presented with various physical or mental disabilities. The beginner riders (BR) consisted of three women and five men aged between 22 and 28 years, who had never ridden a horse before this experiment.

The different parameters measured were chosen for their practicality and feasibility during the trial without interfering with the ridden examination for an excessively long period of time per horse. The parameters were assessed at different time-points before, during or immediately after the session. All parameters, including basal samples, were taken at the same times of the day to rule out any influence of circadian rhythm on secretions (Cordero *et al.* 2012; Diez de Castro *et al.* 2014) and all sampling for analyte concentrations were taken at five-day intervals to avoid any major seasonal interference with the results (Durham *et al.* 2021).

### Session and sampling protocol

Session plans with sampling timings are shown in Table 2. Briefly, the horses were tacked up and taken into the indoor arena by helpers where heart rate and blood samples were obtained (basal samples). Riders mounted their respective horses for a total duration of 1 h, during which time a combination of dexterity pedagogical exercises were performed on their own (no side-walkers were present) under the supervision of the same instructor (GV: 1^st^ degree BEES and Handi’Cheval training diplomate). The horses remained mainly at walk or stood still. A few strides of trot were added towards the end of the session for a maximum duration of 5 min. Horses were brought back to the midline in the arena 20 min after the start of the session, which gave riders sufficient time for a few tasks to be performed, thereby ensuring samples were representative of the ongoing ridden work, before a new blood sample was drawn (T1 samples). Similarly, immediately prior to the end of the session, all horses returned to the midline where a repeat heart rate recording and salivary samples were obtained (T2 samples). Samples were not taken all together in order to limit any disturbance caused to the ongoing session.

**Table 2.** Time-line, description of the ridden sessions and associated samples obtained

Timeline	1130h	1145h	1210h	1220h	1235h	1240h
Riders	Mount	Direction exercises	On track	On track, walk/stop	Dismount	
Horses	Still, midline	Walk	Trot (5 mins)	Walk, still	Still, midline	
Samples	ECG, blood			Blood		ECG, saliva
Associated name of the sample	T0			T1		T2

### Heart rate

**Figure 1.**
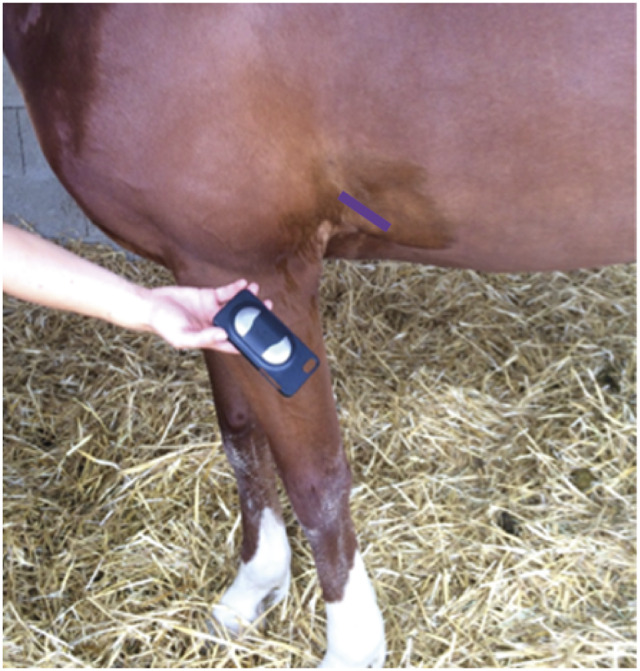
Smartphone case used for ECG recording and the area for correct positioning (purple line).

A smartphone (iPhone 6®, Apple Inc, Cupertino, CA, USA) was used to record an electrocardiogram (ECG) using a case with contact electrodes and an ECG application downloaded from the App Store (AliveCor® Veterinary Heart Monitor, AliveCor Inc, San Francisco, CA, USA). The device has been previously validated for its use in horses with comparable accuracy to a more classic base-apex derivation (Kraus *et al.* 2019). The device was positioned as per manufacturers’ recommendations at a 45° angle behind the left elbow (Figure 1). To improve contact, the skin and coat were dampened with surgical spirit prior to application of the electrodes. The heart rate was evaluated from the trace obtained over a minimum period of 2 min. Close examination of the recording was also performed, including regularity measurements, to detect the presence of arrhythmias.

### Blood analyses: Plasma ACTH and serum total cortisol

Blood samples were obtained via jugular venipuncture using a Vacutainer® system, taken firstly in a tube without anticoagulant and then a tube with ethylene diamine tetra-acetic acid (EDTA) anticoagulant. Less than 4 h post-sampling, both dried and EDTA tubes were centrifuged (2,000g for 10 min and 2,000g for 5 min, respectively) and a minimum of 0.5 mL of serum or plasma were kept in Eppendorf tubes and frozen at –80°C until analysis. All adrenocorticotropic hormone (ACTH) and total cortisol samples were analysed in the same batch, via Chemiluminescence Immuno Assay (Immulite 2000, Siemens Medical Solutions Diagnostics. Erlangen, Germany) with a protocol previously validated for use in horses (Irvine *et al.* 2016; Banse *et al.* 2017).

### Salivary cortisol

**Figure 2.**
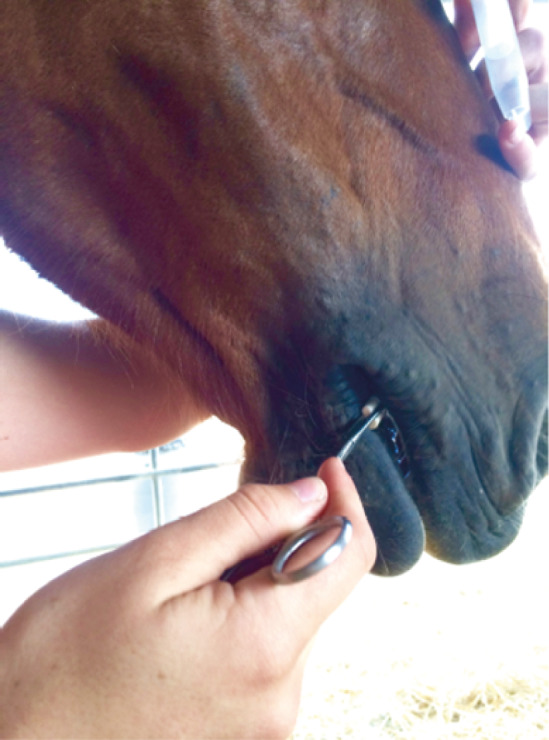
Insertion of a salivette swab in the horse’s mouth using a haemostatic clamp.

Salivettes (Sarstedt, Nümbrecht-Rommelsdorf, Germany) were used to collect saliva using a haemostatic clamp. The salivette swab was placed in the mouth in the interdental space next to the bit when the horse was bitted, or as shown in Figure 2. It was left in place for a few minutes until soaked, depending on the intensity of the horse’s salivation. As these often took a long time to soak (more than 3 min), samples were not taken before the riding sessions (T0) to avoid any delay in the time-scale. Once collected, the salivette swabs were frozen less than 4 h after sampling and until analysis, to help decrease viscosity and remove salivary mucins (Garde & Hansen 2005). On the day of analysis, samples were thawed and at least 1 mL of saliva was obtained via centrifugation at 1,000g for 10 min. An ELISA (enzyme-linked immunosorbent assay) kit, previously validated for use on saliva in horses, was used to measure salivary cortisol (Sauer *et al.* 2020).

### Stress score and behavioural assessment

A digital camera (E-M10 Mark III OM-D, Olympus, Tokyo, Japan) was placed in one corner of the arena, allowing visualisation of more than 70% of the riding area. The entire session was recorded for assessment at a later stage. Each horse’s behaviour was evaluated simultaneously during different time-points when the riders were performing the same exercises (Table 2). Horses’ demeanours were observed over a total length of 15 min taken over three different time-points for each horse: 5 min immediately following the start of the session, 5 min straight after the first sampling, and 5 min following the second sampling. Behaviour was scored as per the specific ethogram established in the pre-clinical study (Table 1), and according to the worse behaviour displayed during the observational time-frame. The final stress score consisted of the mean of the scores obtained following the three separate observations.

### Statistical analysis

The statistical analysis was performed using R software (R Development Core Team 2008). Basal data were examined for normality with the distribution and using QQ plots. The Wilcoxon signed ranks test was used to compare values not normally distributed. Samples obtained at rest were compared to basal T0 samples before IR or BR sessions, when available. This was done to assess the stress levels of horses prior to riding and evaluate if sessions were then going to be comparable. If a significant difference was present, the variation between T1 or T2 values and T0 values were compared between IR and BR groups. If no difference was found, T1 or T2 values were directly compared between IR and BR groups. The investigation of a potential linear correlation between the biological stress markers and the stress score was performed graphically separately for each session, and then using Spearman’s correlation test. A *P*-value < 0.05 was considered to be of statistical significance.

## Results

### Heart rate

No arrhythmias were detected at any point on the recording at rest or during the ridden sessions. Median resting HR at T0 was 35 bpm, not significantly different from the median resting HR of 36 bpm at T2. There was also no significant difference between the median resting HR (35 bpm) and the median value recorded at T0 for HR (37 bpm) and for BR (38 bpm). The median HR with BR at T2 was 38 bpm, no significant difference was found with IR at T2 (39 bpm) or with the resting value at T2. Results are summarised in the first column of Table 3.

### Blood analyses: Plasma ACTH and serum total cortisol

**Figure 3.**
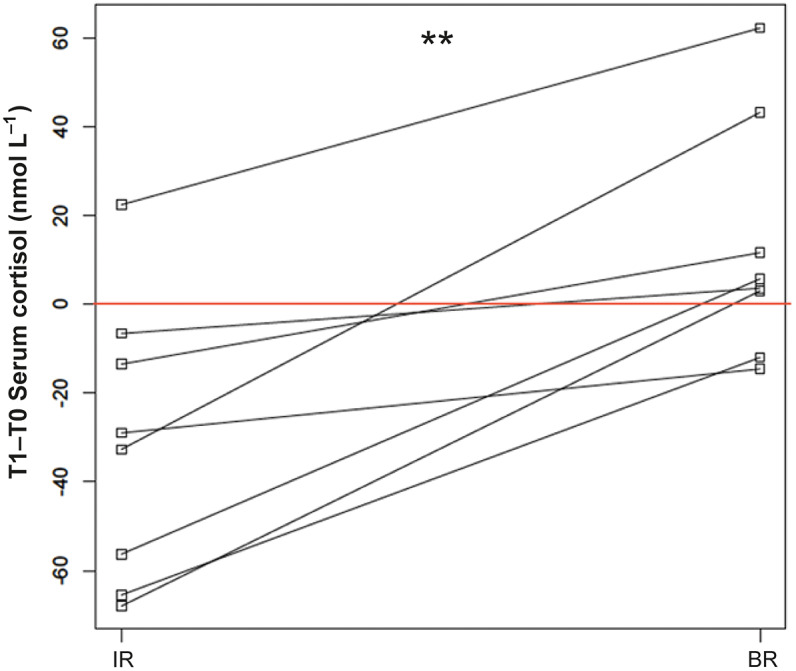
Comparison of serum total cortisol variation for impaired (IR) and beginner riders (BR). ** *P* < 0.01. Lines connect the values obtained for the same horse, red line = no variation observed.

Plasma ACTH results were within the laboratory seasonally adjusted reference intervals, confirming the absence of Pituitary Pars Intermedia Dysfunction (PPID): the median resting ACTH at T0 was 12.5 pg mL^–1^ and the median resting ACTH at T1 was 11.5 pg mL^–1^. Before the ridden sessions, median ACTH values were not significantly different from the resting ACTH and were 13.9 pg mL^–1^ for both IR and BR groups. For the total serum cortisol, the median for IR at T0 was 121.5 nmol L^–1^ which was significantly higher than the resting result 70.4 nmol mL^–1^ (*P* = 0.02) and the BR at T0 81.8 nmol L^–1^ (*P* = 0.05). Considering these and to avoid basal stress levels interference, differences between T1 and T0 for IR and BR were compared rather than values at T1. During the ridden sessions, for IR the median plasma ACTH at T1 was 10.3 pg mL^–1^, which did not differ significantly from BR at T1: 10.5 pg mL^–1^. Comparison of the difference between session and basal values from the same day can be seen in Figure 3. The median variation in serum cortisol with IR (T1–T0) was –30.9 nmol L^–1^, which was significantly lower than for BR at 4.7 nmol L^–1^ (*P* < 0.01). All medians and ranges are shown in Table 3.

**Table 3.** Medians (range) of the physiological parameters obtained from all eight horses at T0 prior to the sessions, and ACTH and serum cortisol at T1 and heart rate, salivary cortisol and stress score at T2

	Heart rate (bpm)	ACTH (pg mL^–1^)	Serum cortisol (nmol mL^–1^)	Salivary cortisol (nmol mL^–1^)	Stress score
Resting T0	35 (30-38)	12.5 (8.6-21.0)	70.4 (45.80-115.0)	1.2 (0.8-2.9)	–
Resting T1/2	36 (28-48)	11.5 (8.7-15.7)	66.6 (32.0-99.3)	1.3 (0.5-7.3)	–
T0 IR	37 (33-60)	13.9 (11.0-32.0)	121.5 (61.8-171.0)	–	–
T0 BR	38 (32-42)	13.9 (5.3-47.7)	81.8 (44.4-103.0)	–	–
T1 IR	–	10.3 (7.2-15.1)	88.3 (48.3-103.0)	–	–
T1 BR	–	10.5 (2.3-19.8)	93.1 (56.0-121.0)	–	–
T2 IR	39 (33-60)	–	–	0.8 (0.1-3.1)	3.0 (0.3-4.3)
T2 BR	38 (28-60)	–	–	1.3 (0.4-4.2)	3.8 (1.7-5.3)

IR = impaired riders; BR = beginner riders.

### Salivary cortisol

**Figure 4.**
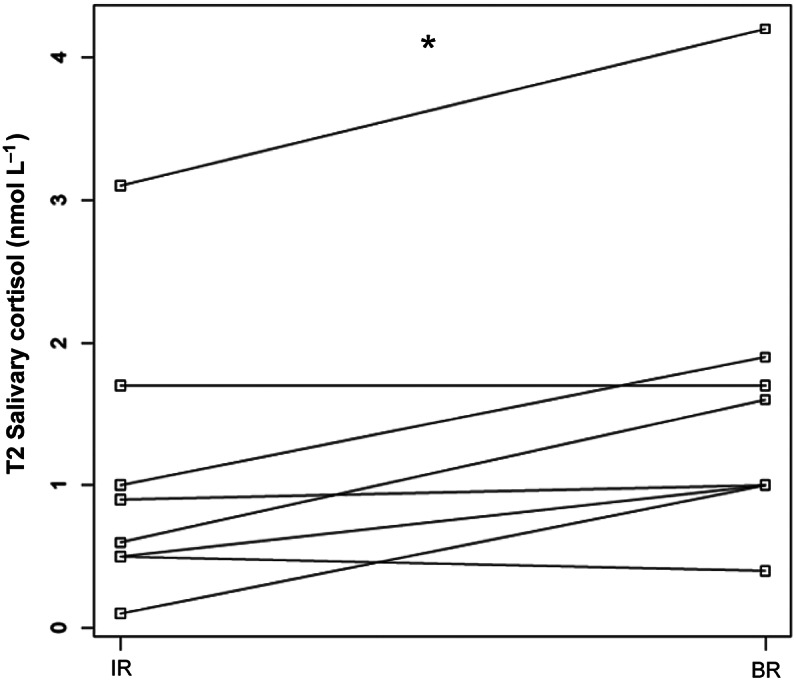
Comparison of the salivary cortisol distributions during the sessions (T2) between the impaired (IR) and the beginner riders (BR). * *P* = 0.04. Lines connect the values obtained for the same horse.

The median resting salivary cortisol concentrations were not significantly different between T0 (1.2 nmol L^–1^) and T2 (1.3 nmol L^–1^). For the values obtained during the sessions, the median salivary cortisol at T2 with IR was 0.9 nmol L^–1^ and with BR it was significantly greater at 1.3 nmol L^–1^ (*P* = 0.04): distributions are shown in Figure 4, medians and ranges in Table 3.

### Stress score

The final stress scores were also not significantly different between BR (score 3.83) and IR (score 3.00) (Table 3). Examination of plotted stress scores in relation to the different biological parameters (including the variation between T1 and T0 serum total cortisol) did not reveal any elliptical distribution which could have evoked a linear correlation (Figures 5[a] and [b]). This was confirmed by the Spearman’s correlation test between stress score and every other parameter which was not significant for any of the parameters analysed separately for both sessions.

**Figure 5.**
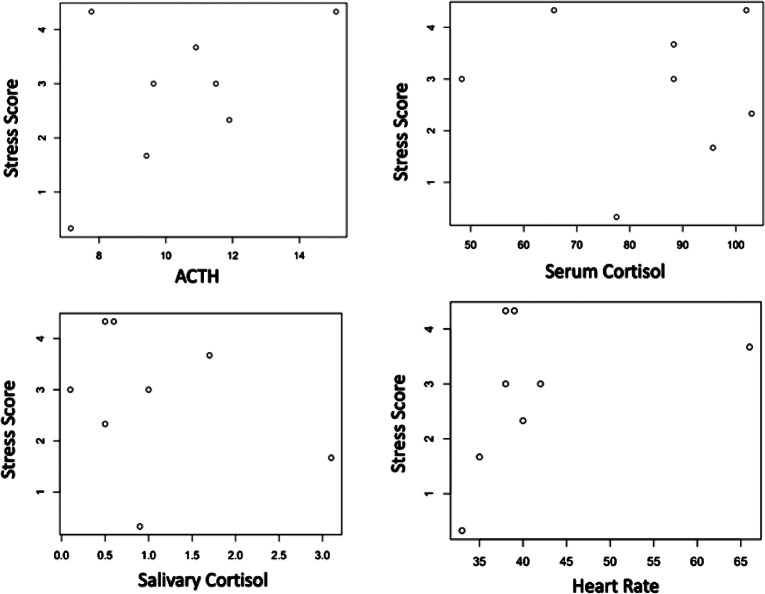
(a) Stress scores plotted according to the related physiological parameter obtained during the IR sessions. No significant correlation was found.

**Figure 5.**
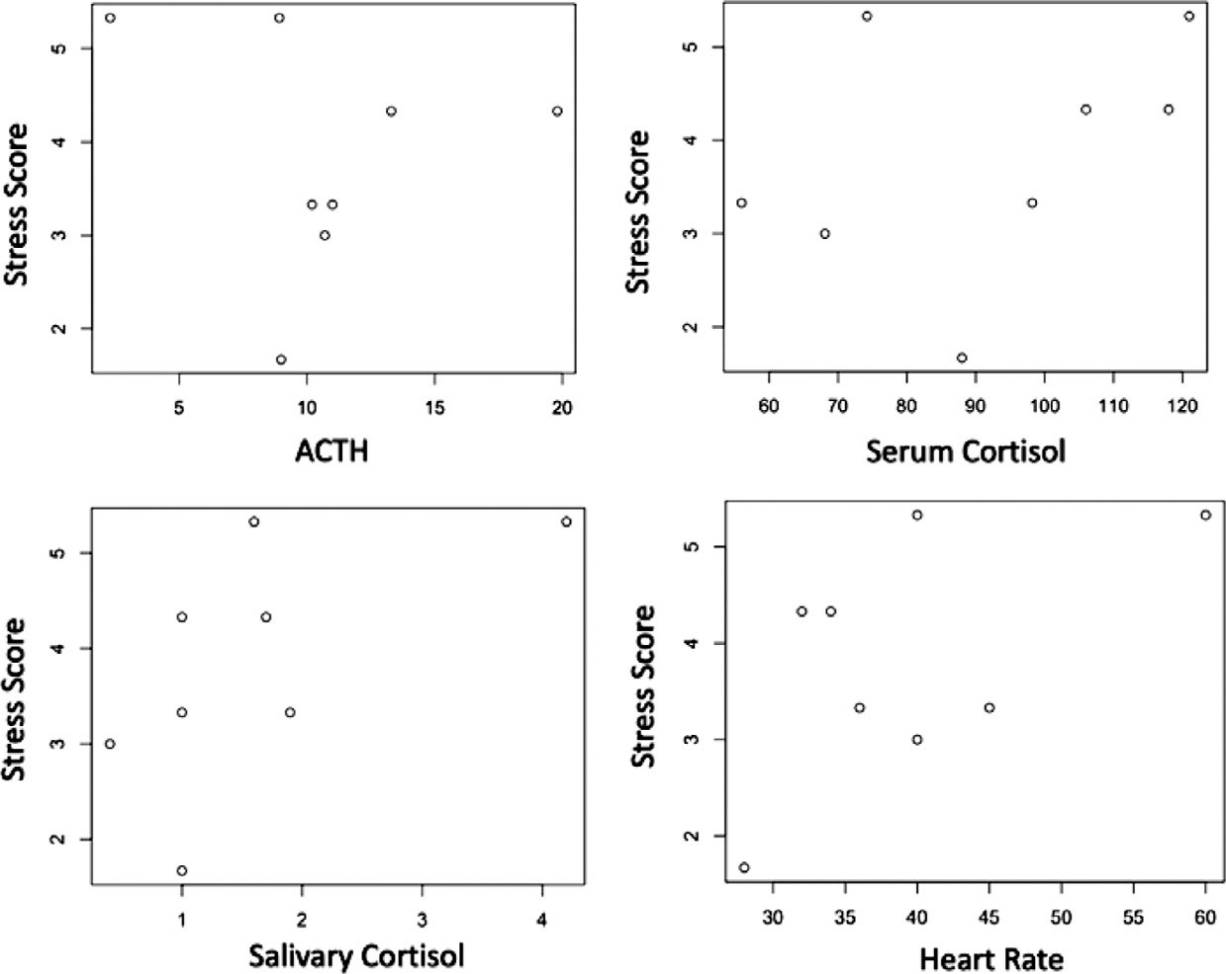
(b) Stress scores plotted according to the related physiological parameter obtained during the BR sessions. No significant correlation was found.

## Discussion

This is one of the first studies to assess both physiological (ACTH, serum and salivary cortisol, heart rate) and behavioural (stress ethogram) parameters at different time-points for horses being ridden by beginners and comparing them to those obtained from horses ridden by disabled people. A significant difference was observed for the serum total cortisol and salivary free cortisol, which were both found to be lower during the hippotherapy session. The serum cortisol was taken 20 min prior to taking the salivary cortisol, a peak in saliva following a serum peak usually by 15 to 20 min (Hurcombe *et al.* 2011). These findings are consistent with a possible reduced systemic stress response during the hippotherapy sessions in comparison with being ridden by beginners, although our sample size means the results should be interpreted with caution.

The main limitation of our study was the small number of horses involved; this is difficult to overcome using a study protocol in the horses’ usual environment since most riding lessons for beginners or hippotherapy sessions tend to take place in an indoor or outdoor arena with limited space. Thus limiting the number of horses involved for obvious safety reasons. In our experiment, the equestrian centre used a maximum of eight horses during all their usual sessions involving beginners or for hippotherapy sessions. The organisation of multicentric experiments or repeated analyses over several weeks could have helped improve the power of our tests if the data could have been assessed and been normally distributed, although significant differences were already found for two of the five parameters examined here. Also, repeating analyses over time can lead to other bias: for example, normal basal ACTH distribution can vary on a weekly basis throughout the year (Durham *et al.* 2021), therefore all measurements in our study were performed within 15 days to limit any such seasonal effect. It is also reasonable to assume that by examining a combination of markers related to stress, the likelihood of finding an abnormality would be greater, especially using physiological parameters which are highly sensitive (Padalino & Raidal 2020). There is also a limitation concerning the use of our ridden ethogram, although based on different published ethograms (Dyson *et al.* 2017; Dyson & Pollard 2020, 2021a,b), the weights that were applied are perfectible and could be improved using horses ridden in different contexts. Adaptation of the grid could be improved by assessing the repeatability, testing for intra- and inter-observer variability, especially with observers familiar and unfamiliar with the grid. Such analyses could lead to the procurement of likelihood of stress from the occurrence of behavioural markers; but the lack of a gold standard method to evaluate the stress status in horses to validate such parameters is still lacking. The nature of the impaired riders’ physical or mental disabilities was not disclosed to us at the time of the experiment to respect the privacy of the riders but this could be investigated more specifically since the influence on the horse could be different. Thus, the use of biological parameters is essential in addition to behavioural analysis, due to the possibility of acquired shorter duration of stress responses (Marsbøll & Christensen 2014).

The sympathetic nervous system and the hypothalamic-pituitary axis are the main actors of biological responses to stressors, where their activation results in adrenal secretions of catecholamines and corticosteroids, respectively (Ayala *et al.* 2012). These messengers modulate important responses, such as heightened alertness, glucose mobilisation/use, vasomotor tone, cardiac output to improve perfusion during times of physiological and pathological stress (Hurcombe 2011). Catecholamines are the first category of biological markers involved in the stress response. Despite their good correlation with systemic stress and sympathetic nervous system stimulation (Wagner 2010), their very short half-life (less than 30 s for adrenaline and noradrenaline) makes them impractical for handling and being measured precisely under field conditions (Snow *et al.* 1992), which is why these analyses did not feature in our study. Tachycardia remains one of the easiest physiological consequences of an increase in catecholamines to observe and was measured precisely via the ECG. Heart rate was found to remain below the expected increase associated with physical stress described in the literature, which lies between 30–50% from the baseline values (Wagner 2010). The ECG also underwent close examination for regularity and complex morphology as the occurrence of premature complexes and runs of tachycardia have been described in relation to stress in horses (Sandersen *et al.* 2005). Heart-rate variability accurately represents the state of stress over an entire session, and this could be used in the future to improve the overall stress assessment (Garcìa-Gòmez *et al.* 2020).

In parallel with the sympathetic-induced responses, inputs also stimulate the hypothalamus which triggers the release of corticotropin-releasing hormone into the pituitary portal system. This causes the rapid release of ACTH by the pituitary gland due to their close proximity. ACTH then binds to adrenal receptors to stimulate (mainly) cortisol release (Hurcombe 2011). All ACTH measurements in our study, especially initial resting samples, were relatively low. They were comprised within seasonally adjusted reference intervals; the experiments being performed in May close to when the yearly ACTH distribution curve shows a natural trough (Durham *et al.* 2021). Elevations above reference ranges are indicative of a systemic stress response, but they may also be influenced by Pituitary Pars Intermedia Dysfunction, a commonly observed endocrine disorder in older horses (Beech *et al.* 2011). Horses were reasonably old in our study (aged between 12 and 21 years) but all resting results were thankfully not consistent with pituitary dysfunction. The aforementioned pathological ACTH elevation is not accompanied by an increased cortisol elevation, which could be due to a compensatory increase in urinary excretion (Morgan *et al.* 2018).

Cortisol, whether in its total or free form found in serum or the free component found in saliva, is a well-used and established stress indicator in horses (Peeters *et al.* 2011; Sauer *et al.* 2019; Ferlazzo *et al.* 2020). The relationship between serum total cortisol, serum free cortisol and salivary cortisol is also intricate and well correlated with stressful stimuli (Alexander & Irvine 1998; Peeters *et al.* 2011; Bohák *et al.* 2013). Reports of the use of salivary cortisol are becoming especially common, essentially because of the ease of sampling and the fact that it is non-invasive (von Lewinski *et al.* 2013; Kang & Yun 2016), and reliable (Garde & Hansen 2005; Sauer *et al.* 2020). Interestingly, during our study sessions, the difference in serum total cortisol and salivary cortisol were the only markers found to be significantly lower during hippotherapy sessions. Baseline cortisol on the day of hippotherapy was significantly greater than on the other days (resting and beginners session day). This prompted us to perform the analysis using the difference between serum cortisol during the session and baseline cortisol from that day. The assumption being that this difference would be a more true reflection of the influence of the session on serum cortisol rather than an initial stress state due to factors external to the sessions. A number of tractors were in use on the yard on the morning of the IR session, but horses were used to occasionally encountering these so there is no certainty about the origin of the baseline cortisol elevation. Although the absence of statistically significant differences among the other parameters does not necessarily mean equivalence, the observations of the distributions were much more homogenous and therefore deemed comparable. In addition, the similar difference observed in the salivary cortisol results support the logic of our analysis. This appears to be in accordance with previous findings in the literature, whereby serum cortisol was equally found to be significantly lower during therapy sessions (Hovey *et al.* 2021) or not affected by contact with people presenting post-traumatic stress disorders (Merkies *et al.* 2018). Other hormonal parameters that could be used to assess stress-coping mechanisms in horses include beta-endorphine and thyroid hormones, which have been studied in healthy horses under different conditions and could add some value to further studies (Ferlazzo *et al.* 2018a,b,c), or oxytocin which has been studied in horses used for post-traumatic stress disorder equine-assisted therapies and activities (Malinowski *et al.* 2018).

The stress ethogram we used did not show a statistically significant difference, nor a correlation with any of the biological parameters measured. However, the overall scores observed during both riding sessions were low (3.83 and 3.00 for the IR and BR, respectively) on a scale that potentially could extent up to 20. This indicates an overall agreement with the low level of stress suggested by the other biological parameters. Developing and validating a stress ethogram in horses is problematic since most of the distressed behaviours expressed can have a subjective interpretation from the observer. Furthermore, it is possible that the focus of horses in accomplishing the task asked by the rider prevents them from expressing otherwise common pain behaviours that could be found at rest (Torcivia & McDonnell 2021). More specifically, it has been shown that horses tend to not express certain discomfort behaviours when reins restrict their movements (Smiet *et al.* 2014). Examples of ethograms developed for ridden work include scales specifically targeting orthopaedic pain (Dyson & Pollard 2020, 2021a,b); these show similarities to our scale although our objective was to investigate discomfort on a more generalised level. The ideal ethogram to assess the latter lies somewhere among all the aforementioned scales. The different weights affected to each parameter need to be established in a more robust mathematical manner, which was beyond the scope of this study.

## Animal welfare implications and conclusion

Our study has shown that within the context of hippotherapy sessions, horses do not present with increased biological stress markers when compared to being ridden by beginners. Furthermore, a statistically significant decrease in cortisol was observed, potentially indicating that being involved in hippotherapy sessions is less stressful for the horses than being ridden by beginners. Although these results indicate that hippotherapy may be ethically justified as it benefits humans without harming the horses, the present study was small, and the results should be interpreted with caution.
